# Editorial: Recent advances and new biomarkers in ulcerative colitis

**DOI:** 10.3389/fmed.2023.1214882

**Published:** 2023-05-30

**Authors:** Tsvetelina Velikova, Xiang Xue

**Affiliations:** ^1^Medical Faculty, Sofia University St. Kliment Ohridski, Sofia, Bulgaria; ^2^Department of Biochemistry and Molecular Biology, University of New Mexico, Albuquerque, NM, United States

**Keywords:** ulcerative colitis, biomarkers, fecal calprotectin, endoscopy, mucosal inflammation, steroid-refractory ulcerative colitis, quality of life, biological therapy

Recent advances in our understanding of ulcerative colitis (UC), one of the two major types of inflammatory bowel disease (IBD), have led to the identification of new biomarkers that may aid in the diagnosis, prognosis prediction, and treatment of this chronic inflammatory condition. Although biomarkers are objective measures of disease activity or response to therapy, it is still a long process to fully use them to guide clinical decision-making. In this editorial, we discuss papers published in the current Research Topic that demonstrate evidence for new biomarkers utilized in experimental UC and patient management.

Experimentally induced models of colitis remain a vital part of the UC research. Yu et al. investigated the GB1a active component of Garcinia Kola nuts as a therapeutic agent for ameliorating experimental UC. They have emphasized the need for biomarkers in the pathways involved in UC pathogenesis, such as NF-κB and Nrf2 signaling pathways. Furthermore, by evaluating the inflammation and oxidative stress through the expression of TNFa-induced inflammatory genes, they suggested that GB1a may regulate inflammation, oxidative stress and permeability (Yu et al.).

One of the most exciting recent advances in UC has been identifying new genetic risk factors for the disease. Genome-wide association studies (GWAS) have identified over 240 genetic loci associated with UC, many of which are involved in the immune response. These genetic findings have led to a better understanding of the underlying pathogenesis of UC. However, the heterogeneity of UC, in terms of disease severity and response to therapy, makes the application of biomarkers very challenging. While some patients have mild diseases that can be easily controlled with medication, others may have severe, refractory diseases requiring more aggressive treatment. Biomarkers that are reliable indicators of disease severity and response to therapy in one patient may not be as reliable in another. One of the most considerable advances is gene signature profiling for predicting- therapy responses in UC and other diseases. Feng et al. demonstrated that an artificial neural network may be used for development of combination random forest to predict primary non-response to infliximab. This study is significant for establishing the role of machine learning in constructing predictive models for therapy responses based on the molecular prognostic score system.

In addition to genetic markers, several new serum and fecal biomarkers have been identified in UC. Serum biomarkers such as C-reactive protein (CRP) and erythrocyte sedimentation rate (ESR) have long been used to monitor disease activity in UC. However, these markers are not specific to UC and can be elevated in various inflammatory conditions. More recently, novel fecal markers (such as calprotectin, lactoferrin) have shown promise as more specific markers of UC activity. The role of fecal calprotectin was confirmed in 143 patients with UC by Chen et al., establishing a level of 164 μg/g as the level with 85.42% sensitivity and 73.68% specificity in predicting clinically active disease and mucosal healing at 154.5 μg/g with a sensitivity of 72.34% and specificity of 85.71%. Other fecal biomarkers such as M2-PK, and S100A12 have also shown promise as markers of UC activity but are less investigated. Shi et al. presented evidence from published systematic reviews and meta-analyses in their umbrella review. Markers, such as anti-neutrophil cytoplasmic antibodies, anti-neutrophil cytoplasmic antibodies, and imaging techniques (i.e., ultrasound and magnetic resonance enterography) validated their role for assessing disease activity (Shi et al.). This is also valid for other promising markers, such as trefoil factor 3 (1), which correlates with disease activity and predicts complete mucosal healing. Mucosal healing could be predicted by assessing mucosal vascular patterns under special imaging endoscopy called narrow-band. He et al. demonstrated that narrow-band imaging endoscopic staging of mucosal vascular patterns could predict histological healing and clinical recurrence of UC.

Less employed are the biomarkers in other biologic samples, such as urine. However, Gunawan et al. showed that urinary chemerin is a promising non-invasive marker for monitoring UC severity and clinical course. Other bright research fields not covered in current state-of-the-art papers collection are gut microbiome biomarkers.

Prediction of therapy response is one of the ultimate goals when discussing the effectiveness and safety of biological therapy for UC. As Zhou et al. showed in 146 patients with UC, the novel biomarker, the neutrophil-to-albumin ratio, was positively associated with the disease activity. Moreover, this ratio could discriminate initial responders to primary non-responders to infliximab induction therapy. Thus, it could be employed in diagnosing, monitoring and predicting treatment efficacy in UC patients (Zhou et al.).

In their systematic review and meta-analysis, Szemes et al. shed light on evaluating the long-term outcomes of cyclosporin and infliximab in steroid-refractory UC patients. Since they did find significant differences for colectomy-free survival in favor of infliximab in the first 3 years, no long-term differences were observed for severe adverse events or deaths. From all the 15 studies analyzed in the meta-analysis and 1,607 patients with steroid-refractory acute severe UC, authors did not find definitive evidence for any differences in cyclosporin and infliximab efficacy and safety in patients with severe acute UC (Szemes et al.).

The quality of life is part of UC patients' integrative management approach. Li et al. constructed a prediction model for IBD, focusing mainly on the factors that affect the quality of life, especially emotional function and systemic symptoms. Interestingly, annual household income, occupational stress and score on the IBD questionnaire were independent risk factors for UC recurrence.

In conclusion, recent advances in our understanding of UC have led to identifying new biomarkers that may aid in diagnose, prognosis prediction, and disease treatment. As our knowledge of the underlying pathogenesis of the heterogenous UC continues to evolve, we will need to address significant challenges such as low specificity, sensitivity and cost to extend the usefulness of new biomarkers in the clinical practice. Standardizing biomarker assays and developing clear guidelines will increase the reliability of these biomarkers. This requires validation in larger, multicenter studies, including a more diverse patient population.

## Author contributions

Both authors listed have made a substantial, direct, and intellectual contribution to the work and approved it for publication.
